# Feeding Date-Palm Leaves Ensiled with Fibrolytic Enzymes or Multi-Species Probiotics to Farafra Ewes: Intake, Digestibility, Ruminal Fermentation, Blood Chemistry, Milk Production and Milk Fatty Acid Profile

**DOI:** 10.3390/ani12091107

**Published:** 2022-04-25

**Authors:** Ahmed E. Kholif, Hatem A. Hamdon, Gouda A. Gouda, Ayman Y. Kassab, Tarek A. Morsy, Amlan K. Patra

**Affiliations:** 1 Dairy Science Department, National Research Centre, 33 Bohouth St. Dokki, Giza 12622, Egypt; gagouda@gmail.com (G.A.G.); tareknrc@yahoo.com (T.A.M.); 2 Department of Animal Production, Faculty of Agriculture, New Valley University, El-Kharga 72511, Egypt; hamdon@nv.aun.edu.eg (H.A.H.); ayman15@yahoo.com (A.Y.K.); 3 Department of Animal Nutrition, West Bengal University of Animal and Fishery Sciences, 37 K.B. Sarani, Kolkata 700037, India; patra_amlan@yahoo.com

**Keywords:** date palm leaves, ensiling, fibrolytic enzyme, lactic acid bacteria, feed utilization, milk production, multi-species probiotics, ruminal fermentation

## Abstract

**Simple Summary:**

Under semi-arid and arid conditions, trees and shrubs such as date palm can be used as an adequate source of feed for goats and sheep to reduce feed cost. However, the low nutritive value of such materials determines its nutritive value. Ensiling with fibrolytic enzymes or lactic-acid bacteria can be used to enhance the nutritive value of date palm leaves and other agricultural byproducts before feeding to animals. Exogenous enzymes can alter the structure of the tissue while lactic-acid bacteria improve ensiling in enhancing nutrient digestibility, resulting in improved performance (daily gain or milk production). This may enhance farmers’ gain and animal health. This is the first experiment to utilize ensiling with fibrolytic enzymes or lactic-acid bacteria to enhance the nutritive value of date palm leaves as an unconventional feed.

**Abstract:**

The present experiment evaluated the feeding of date palm leaves (DPL) ensiled with fibrolytic enzymes (ENZ) or multi-species probiotics (MSP) on nutrient utilization and lactational performance of ewes. Fifty multiparous lactating Farafra ewes were used in a completely randomized design for 90 d. The treatments consisted of the control diet with a concentrate feed mixture and date palm leaves (at 60:40, DM basis) ensiled without additive (control) or DPL ensiled with ENZ or MSP replacing control DPL at 50 or 100%. Both ENZ and MSP increased (*p* < 0.01) DPL and total intakes, digestibility of all nutrients, concentrations of ammonia, total volatile fatty acids, acetate and propionate in the rumen. Increased milk production, concentrations of fat, lactose and energy in milk, and feed efficiency were observed with MSP and ENZ compared to the control treatment. Moreover, ENZ and MSP increased (*p* < 0.05) the concentrations of total n3, n6 fatty acids, polyunsaturated fatty acids and conjugated linoleic acids and decreased (*p* < 0.001) the atherogenicity. The differences between ENZ and MSP and between the low and high replacement levels were minor for all measured parameters. Ensiling of DPL with MSP or fibrolytic enzymes is recommended to improve feed efficiency and improve lactational performance of ewes.

## 1. Introduction

In semi-arid and arid regions, improvement of utilization of available feed resources and search of alternative feeds for ruminants are required due to the shortage of green fodders. Egypt and many other countries lack adequate availability of animal-feed ingredients, causing the utilization of unconventional feeds and secondary agricultural products as a premium approach to feed animals. However, most of the unconventional feeds have a limited nutritive value and some improvements should be considered before feeding to ruminants to obtain optimum production performance.

Date palm (*Phoenix dactilifera*) is one of the main crops in Egypt and many semiarid and arid regions of the world. In Egypt, there are around 650,000 tons of leaves’ dry matter (DM) available from date palms annually [1], but without significant utilization. The main problem with the date-palm leaves (DPL) is the high fiber and low crude protein (CP) content and low nutritive value and digestibility causing its limited utilization as a feed for ruminants. The CP content in DPL ranges from 42 to 165 g/kg DM [1,2]. Fiber content in DPL is high, ranging from 430 to 730 g/kg DM of neutral detergent fiber (NDF) [1,2]. Therefore, improvement of its nutritive value is recommended before feeding to ruminants.

Ensiling is a good approach to improve the nutritive value of poor-quality forages [3] and reduces the negative effects of some antinutritional factors present in feeds [1]. To improve the ensiling process, some inoculants and other feed additives are included during the ensiling process to improve the anerobic conditions and fermentation of silage [4]. Multi-species probiotics (MSP) such as lactic-acid bacteria (LAB) [5,6], and fibrolytic enzymes [3] are good examples of these feed additives. Inoculating silages with MSP improves silage characteristics and prohibits the growth of undesirable bacteria and other spoilages, and increases the initial LAB growth in silages [4]. Kaewpila et al. [6] stated that inoculating forage sorghum mixture silage with LAB could promote ensiling characteristics (e.g., lowering pH and increasing lactic-acid contents) and nutritive value (increased in vitro DM degradability and total gas production, and decreased methane production). Lactic-acid bacteria also improve gut health, immunity and productive performance of animals [7,8], which will be added to advantages besides improving silage fermentation [8]. In the study by Hamdon et al. [7], Farafra lambs fed DPL-based diets supplemented with MSP showed higher growth performance, feed intake and feed efficiency. In another experiment, Maake et al. [9] reported that feeding MSP to South African goats had no effect on feed intake, but increased average weight gain.

The administration of fibrolytic enzymes during ensiling was reported to improve nutritive value of feeds, especially those with low nutritive value such as agricultural byproducts [3,10]. Mixing the enzymes into the diet prior to feeding is the most effective way to maximize their nutritive potential [11]. Administration of enzymes prior to feeding (e.g., during ensiling) allows enzymes to attach to the target nutrients (especially those related to fiber components) before consumption, causing a reduction in the lag time between consumption and ruminal degradation. Additionally, administration of fibrolytic enzymes in solutions before ensiling improves the enzyme function due to the hydrolysis of soluble sugars (i.e., glucose) from a complex polymer (such as cellulose). This process of hydrolysis involves the addition of water to specific bonds within a complex carbohydrate, and can be limited if there is insufficient water in the environment [11]. Fibrolytic enzymes also alter ruminal fermentation characteristics and increase fiber digestibility through solubilizing dietary fiber components [11]. Moreover, fibrolytic enzymes increase the supply of readily fermentable nutrients to ruminal microorganisms, and increase the microbial enzyme activities and microbial attachment to feed particles in the rumen [12]. Recently, Abid et al. [10] evaluated the enzymatic treatment of olive-mill waste containing a high lignocellulose content and high concentrations of anti-nutritional factors with exogenous fibrolytic enzymes produced from *Trichoderma longibrachiatum* as a feed for ruminants. They observed that enzymatic treatments increased degradation of cellulose and hemicellulose and increased the amounts and rate of gas production as well as the microbial crude protein production. Azzaz et al. [13] observed that feeding lactating goats on diets supplemented with fibrolytic enzymes improved feed utilization, milk production, and composition and feed efficiency.

We hypothesized that inoculating DPL during silage-making with fibrolytic enzymes would help in degrading the rigid structure of fibers in DPL, making the nutrients available for animals. Moreover, we hypothesized that inoculating silage with MSP containing LAB, along with other probiotic bacteria such as *Bacillus subtilis* and *Bacillus lichenifomis* would facilitate maintenance of the ensiling conditions and improve the nutritive value of ensiled DPL before feeding. The present study aimed to evaluate the effects of inoculating DPL with MSP or fibrolytic enzymes during ensiling on its nutritive value as a feed for lactating Farafra ewes under the arid conditions in the New Valley area in Egypt.

## 2. Materials and Methods

### 2.1. Study Location

This experiment was carried out at the experimental farm of the Department of Animal Production, Faculty of Agriculture of New Valley, Al Kharga, Egypt (25°26′ N and 30°32′ E). The chemical analyses were performed at the laboratory of Dairy Animal Production, National Research Centre, Cairo, Egypt. Animals were managed and cared for in accordance with the 3rd edition (2010) of the guide of the Agricultural Research and Teaching of Federation of Animal Science Societies, Champaign, IL, USA. The protocol of the experiment was revised and approved by the Institutional Animal Care and Use Committee of the Faculty of Agriculture, New Valley University, New Valley, Egypt.

### 2.2. Date-Palm Leaves

Fresh DPL were collected from different sites in the New Valley Governorate (Egypt). Materials were sun-dried for 10 days [7]. Date-palm leaves were ensiled under anaerobic conditions for 45 days using tightly closed polythene bags. Briefly, the chopped DPL were spread with a solution containing clean water and solid urea (40 g/L solution) and crude liquid molasses (40 g/L solution). Before ensiling, moisture content in DPL was increased to reach about 35–40% with the urea-molasses solution. Three types of DPL were prepared: DPL ensiled without fibrolytic enzymes or MSP and DPL ensiled with fibrolytic enzymes (Polyzyme, Zeus Biotech, Mysuru, India) at 4 g/kg DM or MSP (ProAct, Bengaluru, India). The materials were packed into polythene-bag silos (40 × 70 cm) and compressed manually to create an anaerobic environment. The enzyme product contained (per kg): 4 × 10^6^ IU xylanase, 4 × 10^5^ IU cellulase, 2.4 × 10^5^ IU pectinase, 2 × 10^5^ IU β-glucanase, 21.5 × 10^6^ IU amylase, 7.5 × 10^5^ IU protease, 4 × 10^5^ IU galactosidase, 2 × 10^5^ IU mannanase, 5 × 10^4^ IU phytase and 4 × 10^4^ IU lipase along with fermented rice bran. In addition to some species of LAB, the MSP contained 1.75 × 10^12^ CFU *B. subtilis* and 1.75 × 10^12^ CFU *B. lichenifomis* per gram product and dextrose monohydrate as a filler.

For assessment of the ensiling process, 200 g (fresh weight of silage was mixed with 800 mL distilled water, homogenized for 3 min with a blender and filtered through 4-layer cheesecloth. The filtrate was collected for measurement of pH using a digital pH meter, ammonia-N (NH_3_-N) according to AOAC [14], and volatile fatty acids (VFA) according to AOAC [14]. Aflatoxin F_1_ concentration was measured in silage with the use of a Fluorometer, Series-4 (Vicam, Milford, MA, USA) based on the methods described by AOAC [14].

### 2.3. Ewes and Management

Two weeks before expected parturition, fifty lactating Farafra ewes (mean ± standard deviation: 2 ± 1.2 parity; 33.3 ± 3.04 kg body weight; 24 ± 3.3 months of age; 550 ± 10/4 g/d of previous milk production) were assigned randomly to five dietary treatments (*n* = 10 ewes/treatment). Ewes were randomly stratified to treatments in a completely randomized design. Ewes were individually kept in semi-opened concrete floor pens (1.5 m^2^/sheep) with free choice fresh water. Sheep were fed a diet comprising (per kg DM) 600 g of a concentrate feed mixture and 400 g of DPL ensiled without additives in the control treatment. In the other four diet treatments, ensiled (without fibrolytic enzymes of MSP) DPL of the control treatment diet was replaced with DPL ensiled with fibrolytic enzymes (ENZ50 and ENZ100) or MSP (MSP50 and MSP100) at 50 or 100% level, respectively. Ewes were first offered the allotted amounts of concentrate feed mixture in the feeder, followed by DPL after the consumption of concentrate feed. Diets were prepared to meet nutrient requirements for milk production of ewes according to NRC [15] recommendations. To ensure orts collection, feeds were offered 1.10 times above the NRC recommendations. The experiment lasted for 90 d. Individual animals were weighed at monthly intervals. Table 1 shows the chemical compositions of ingredient and experimental diets. The daily samples of diets were composited weekly and dried at 60 °C in a forced-air oven for 48 h [14] (method 930.15) before storing for chemical analyses.

### 2.4. Feed Intake and Nutrient Apparent Digestibility

Three digestibility trials were conducted during the last 10 d of each month using acid-insoluble ash as an internal indigestibility marker. The equations of Ferret et al. [16] were used to calculate the coefficients of apparent digestion. Feed intake was calculated as the difference between feed offered and orts from the previous day’s feeding. Individual fecal grab samples were collected twice daily during the collection period at 07:00 and 15:00 h, dried at 60 °C in a forced-air oven for 48 h, and pooled per ewe.

Composited samples of dried feeds, orts and feces were ground to pass through a 1mm screen using a mill and analyzed for DM, ash, nitrogen, and ether extract (EE) according to AOAC [14] official methods. Neutral detergent fiber and lignin contents were determined according to Van Soest et al. [17]. Acid detergent fiber (ADF) content was analyzed according to AOAC [14] and expressed exclusive of residual ash. Non-structural carbohydrates, cellulose, hemicellulose, and organic matter (OM) concentrations were calculated.

### 2.5. Sampling and Analysis of Rumen Fluid

On d 30, d 60 and d 90 of the experiment, ruminal fluid samples were collected from all animals in the morning at 3 h post feeding to analyze fermentation variables (VFA and NH_3_). About 100 mL of ruminal fluid was collected from each ewe and strained through 4 layers of cheesecloth for NH_3_-N analysis [14] and VFA [18] determination. The collected samples were preserved at −20 °C pending analyses. Concentration of VFA and its individual molar proportions were determined using a gas chromatograph (Thermo Fisher Scientific, Inc., TRACE1300, Rodano, Milan, Italy) fitted with an AS3800 autosampler and equipped with a capillary column HP-FFAP (19091F-112; 0.320 mm o.d., 0.50 μm i.d., and 25 m length; J & W Agilent Technologies Inc., Palo Alto, CA, USA). A mixture of known concentrations of individual short-chain fatty acids was used as an external standard (Sigma Chemie GmbH, Steinheim, Germany) to calibrate the integrator.

### 2.6. Sampling and Analysis of Blood Serum

On d 30, d 60 and d 90 of the experiment, blood samples (10 mL) were collected at 4 h post feeding from the jugular vein of each ewe into clean dry tubes without anticoagulants. Collected samples were centrifuged at 4000× *g* for 20 min at 4 °C, and serum was decanted into 2-mL Eppendorf tubes and frozen at −20 °C pending analysis using specific kits (Stanbio Laboratory, Boerne, TX, USA) according to manufacturer instructions. Globulin concentration was calculated (total protein—albumin).

### 2.7. Milk Sampling and Composition

Ewes were hand-milked during the last 10 d of each experimental period at 09:00 and 21:00 h, and 10% of recorded milk yield samples was taken at each milking and composited daily for the analysis of milk components (fat, lactose, total solids, and protein) using infrared spectrophotometry (Lactostar Dairy Analyzer, Funke Gerber, Berlin, Germany).

Fatty acids in milk were determined using methyl esters prepared by base-catalyzed methanolysis of the glycerides (potassium hydroxide in methanol) according to International Standards (International Standard ISO 15884-IDF 182. 2002, Brussels, Belgium: International Dairy Federation) on a Perkin-Elmer chromatograph (model 8420, Beaconsfield, Perkin Elmer, Beaconsfield, UK) equipped with a Cp-Sil 88 fused-silica capillary column (100 m × 0.25 mm internal diameter × 0.2 µm film thickness; Chrompack, Middelburg, Netherlands) and a flame ionization detector (Perkin Elmer, Beaconsfield, UK). Atherogenic index (AI) was calculated according to Ulbricht and Southgate [19].

Gross energy content in milk, fat-corrected milk (4% FCM, kg/day) and energy-corrected milk (ECM, kg/day) were calculated according to Tyrrell and Reid [20]. Feed efficiency was calculated and expressed as milk yield, FCM, and ECM per unit of DM intake.

### 2.8. Statistical Analyses

The Shapiro-Wilk test was used to test the normal distribution of data. For the small number of variables that showed significance for the Shapiro-Wilk test, data transformation (e.g., natural log, inverse of the natural log, square root, or inverse of the square root) was applied before statistical analysis. Data were analyzed using a completely randomized design with repeated measurements in time, in which each ewe was an experimental unit using PROC MIXED of SAS (Online Version, SAS^®^ On Demand for Academics, SAS Inst., Inc., Cary, NC, USA). The following model was used as:

Y_ijkl_ = μ + T_i_ + A_j_(T_i_) + P_k_ + (T × P)_ik_ + E_ijkl_ (1)

where Y_ijkl_ expressed each observation of the *j*th ewe in the *k*th sampling time given *i*th diet, T_i_ expressed the diet’s effect, A(T)_ji_ expressed the ewe within each diet, P_k_ expressed the sampling week effect, (T × P)_ik_ expressed the interaction between the diets and sampling period, and E_ijkl_ expressed the experimental error. Polynomial (linear and quadratic) contrasts were used to examine level responses to increasing the level of DPL separately for enzyme or MSP effect. Additionally, contrast between enzyme vs. MSP treatments was applied. The period and diet × period interactions were non-significant (i.e., *p* > 0.05) for most of the measurements; thus, only the main effects of diets were reported. Significance was declared at a level of *p* < 0.05.

## 3. Results

### 3.1. Feed Intake and Apparent Nutrient Digestibility

Without differences between enzyme and MSP treatments, ensiling of DPL with enzymes or MSP increased (linear and quadratic effects, *p* < 0.01) both DPL and total intakes compared to the control ewes (Table 2). Feeding DPL ensiled with MSP or enzymes increased (*p* < 0.01) the digestibility of nutrients linearly (all nutrients) and quadratically (all nutrients except for NDF for MSP treatment and EE for both enzyme and MSP treatments). The digestibility of DM was greater for MSP vs. enzyme (*p* = 0.001), whereas NSC digestibility (*p* = 0.005) and cellulose digestibility (*p* = 0.029) were greater for enzyme compared with the MSP treatment.

### 3.2. Ruminal Fermentation

No differences were observed between MSP and enzyme treatments for all measured ruminal parameters except for an NH_3_-N concentration that was higher (*p* = 0.029) for MSP treatment than enzyme treatment (Table 3). Without affecting ruminal pH, the butyrate concentration or acetate:propionate ratio, enzyme and MSP treatments increased (linear *p* < 0.001 and quadratic effects *p* < 0.05) the concentrations of ruminalNH_3_-N, total VFA, acetate and propionate.

### 3.3. Blood Chemistry

Treatments did not affect serum globulin, urea-N, aspartate aminotransferase (AST), alanine aminotransferase (ALT), low density lipoprotein (LDL), high density lipoprotein (HDL), and beta-hydroxy butyric acid (BHBA) concentrations (Table 4). Both enzyme and MSP treatments increased (linear and quadratic effects, *p* < 0.01) the concentrations of serum total protein, albumin, glucose and antioxidant capacity. Concentration of non-esterified free fatty acid (NEFA) increased linearly with increasing PDL level treated with MSP. No differences between MSP and enzyme treatments were noted for most of the variables except for urea-N and NEFA that were greater for MSP treatment than for enzyme treatment.

### 3.4. Milk Yield, Composition and Fatty Acids

There were no differences between enzymes and MSP treatments for milk production and yields of milk components. However, DPL ensiled with enzymes or MSP linearly increased (*p* < 0.01) the production of milk, ECM, FCM, and yields of milk components (Table 5). Without affecting milk protein and ash contents, enzymes and MSP treatments increased (linear *p* < 0.001, and quadratic effects, *p* < 0.01) the concentrations of milk total solids, solids not fat, fats, lactose, and milk energy. MSP treatments showed higher (*p* < 0.001) contents of solids not fat and lactose compared to enzyme treatments. Moreover, treatments linearly improved (*p* < 0.05) feed efficiency calculated as milk:intake, ECM:intake or FCM:intake ratios.

### 3.5. Milk Fatty Acids

Both enzyme and MSP treatments increased (linear and quadratic effects, *p* < 0.05) the concentrations of C18:1n9 *trans* (linear effect), C18:2 *trans*-10, *cis*-12, C18:3n3, C18.3n6, C20:5n3, polyunsaturated fatty acids (PUFA), total conjugated linoleic acids (CLA), and total unsaturated fatty acids (UFA) to total saturated fatty acids (SFA) ratio, while they decreased (linear and quadratic effects, *p* < 0.001) the atherogenicity index (Table 6). No differences were observed between enzymes and MSP treatments for all detected fatty acids in milk except for C14:0 (*p* = 0.044).

## 4. Discussion

### 4.1. Feed Intake and Nutrient Apparent Digestibility

Fibrolytic enzymes and MSP increased DPL intake by 7.3, 8.6, 7.1 and 8.7%, for ENZ50, ENZ100, MSP50 and MSP100 treatments, respectively compared to the control treatment indicating improved palatability with the fibrolytic enzymes and MSP addition. The improved nutrient digestibility and ruminal fermentation with treatments are another probable reason for greater feed intake. Increasing the rate of fermentation of the insoluble fraction following the use of fibrolytic enzymes, and MSP may reduce the rumen fill and consequently increase the feed intake of low-energy feed [10]. Moreover, the administration of enzymes during ensiling allows enzymes to be quickly attached to fiber and reduces the lag time resulting in increasing feed intake and digestibility [11].

It is well-documented that DPL contain high concentrations of various plant secondary metabolites such as tannins, and flavonoids, waxes, isoflavones, and lignans, which can adversely affect nutrient digestibility [21]. In the present experiment, fibrolytic enzymes and MSP improved all nutrient digestibility, with different modes of action. Improved ruminal fermentation with MSP and fibrolytic enzymes may result from greater nutrient digestibility, particularly the fiber fractions. Hamdon et al. [7] reported that DPL have low nutrient digestibility when wheat straw was replaced by DPL due to the high fiber concentration in DPL. In the present experiment, the improved CP and cellulose digestibility indicates that ensiling with the additives (enzymes and MSP) may loosen the association among the fiber bundles and between protein and fiber fractions in DPL [7]. The highest digestibility of DM and cellulose were observed with MSP50 and MSP100 treatments compared to other treatments, which indicate better rumen fermentation rates and patterns with MSP treatment [8]. Although activity of ruminal cellulolytic microbial populations was not measured in the present experiment, MSP supplementation could produce a tonic level of lactate, which would then boost a basal abundance of lactate utilizing bacteria in the rumen, thus stabilization and fluctuation of pH [22,23]. The improved ruminal environment may increase the fiber-degrading microbial communities in the rumen, resulting in improved nutrient digestion and synthesis of microbial proteins [24]. Moreover, the increased digestibility with MSP treatment may improve the interaction of MSP with the ruminal microbial flora [9,25]. Generally, probiotics may increase enzyme activity in the gastrointestinal tract and improve digestibility [8,26].

The higher digestibilities of nutrients with fibrolytic enzymes’ treatment are some of the advantages of adding exogenous enzymes in ruminant diets. Exogenous fibrolytic enzymes can break the cross-linkages between cell wall substances and lignin, and solubilize cell-wall components (mainly hemicellulose) [3]. Moreover, fibrolytic enzymes might cause some changes in the rate of potentially degradable NDF in the rumen [27] and the activity and number of ruminal non-fibrolytic and fibrolytic microbiota [12]. Fibrolytic enzymes increase the ruminal degradability rate of the potentially digestible NDF [27], changing the nutrient digestibility site, enhancing ruminal microbiota attachment and plant cells colonization and the synergy between exogenous enzymes and ruminal endogenous enzymes and microflora [12,28]. Ensiling of DPL with fibrolytic enzymes may cause hydrolysis of complex carbohydrates into simple sugars that may be utilized by ruminal microbiota for growth and stimulating more microbial growth, resulting in changes in the overall rumen microbial population and enzymatic activity in the rumen [11].

### 4.2. Ruminal Fermentation

Both MSP and fibrolytic enzymes have almost the same effects on ruminal fermentation. Treatments did not influence ruminal pH values that were greater than the optimum level (5.6) for ruminal fiber degrading microbial activities and growth [29]. Preventing a decline in ruminal pH is important to avoid a change in ruminal microbiota from predominantly fibrolytic to amylolytic microbial communities [30]. Fibrolytic enzymes and MSP treatments increased the concentrations of ruminalNH_3_-N, which may be a result of the increased CP digestibility. The observed NH_3_-N concentrations ranged between 30.1 to 32.9 mg/dL, which was greater than the level (8.5 to over 30 mg NH_3_-N/dL) for optimum rumen microbial proliferation and activity [31].

The increased concentrations of ruminal total VFA, with fibrolytic enzymes and MSP treatments, may be attributed to improved nutrient digestibility (e.g., organic matter and NSC digestibility). Increased total and individual VFA concentrations of the enzymes- and MSP-treated diets likely resulted from the increased feed intake, N and fermentable carbohydrate availability, ruminal microbial activity and fermentation rate. Similar results were observed by Abid et al. [10]. Additionally, the treatments increased the concentrations of ruminal acetate with fibrolytic enzymes and MSP, which could be the result of improved apparent fiber degradation [5]. Fibrolytic enzymes and MSP treatments increased the ruminal propionate concentrations, which may result from improved apparent nutrient degradation, especially NSC digestibility by ruminal enzymes. Treatment of DPL with MSP generally improved ruminal fermentation in vitro when berseem hay was replaced with MSP-treated DPL at a 25% level [5].

### 4.3. Blood Chemistry Measurements

In the present experiment, all the measured serum biochemical variables were within the standard physiological ranges for healthy ewes, indicating good health with normal nutritional and physiological status of the ewes. Treatments did not influence the concentrations of serum globulin or urea-N, indicating minimal effects of treatments on ewes’ nutritional status, muscle protein catabolism and unaltered kidney function [32]. Moreover, treatments did not affect the concentrations of serum ALT or AST, suggesting minimal effects of treatments on liver health [33]. Hamdon et al. [7] observed that feeding DPL to ewes increased serum ALT levels. The unaffected concentrations of serum HDL or LDL with treatments indicate the uninfluenced treated DPL on fat metabolism, liver dysfunction, and fat malabsorption [34]. Additionally, the unchanged or minor changes in concentrations of serum NEFA and BHBA indicate that body-fat breakdown was not changed and the ewes were not in a negative energy balance in the DPL treatments [35].

Both the MSP and enzyme treatments increased the concentrations of serum total protein and albumin, which are important indicators for improved nutritional and physiological status of the ewes due to increased nutrient intake and digestibility. Increased serum total protein and albumin can be related to higher feed intake and nutrient supply in the ewes fed treated DPL. Additionally, MSP and enzyme treatments increased the concentrations of serum glucose, which may be associated with the observed enhanced apparent organic matter and NSC digestibilities. Serum glucose concentration has a strong relationship with the concentration of ruminal propionate that increased in the present study due to enzymes and MSP treatment of DPL because blood glucose is synthesized from ruminal propionate in the liver [36]. This result, additionally, corroborates with the unchanged NEFA and BHBA concentrations in serum that ewes in DPL treatments were not in deficient energy balance despite greater milk production in these groups. Although ruminal NH_3_-Nconcentration elevated with the additive use, this was not reflected in blood urea-N concentration. Blood urea-N in ruminants is a function of several factors, including absorption of ruminal NH_3_-N, efficiency of utilization of absorbed amino acids, catabolism of protein, and transfer of blood urea to milk and its excretion rate.

Increasing the antioxidant capacity in ewes fed diets treated with MSP and enzymes is paralleled with the results of Sharifi et al. [21], who observed that feeding low-quality date palm to lactating goats improved total antioxidant capacity in milk and blood. Ensiling of phenolic-rich leaves increased the concentrations of phenolic acids and flavonoid compounds that have antioxidant properties. The antioxidants and phenolic compounds in DPL, which may be more available due to treatments with MSP and enzymes than the untreated DPL, may increase antioxidant status in blood.

### 4.4. Milk Yield, Composition and Fatty Acids

Fibrolytic enzymes and MSP treatments increased the daily production of milk (9, 10.7, 8.1 and 9.5%), ECM (15.3, 17.5, 14.7 and 16.6%), and FCM (15.6, 18.1, 14.2 and 15.6%) for ENZ50, ENZ100, MSP50 and MSP100 treatment, respectively. Increasing milk production in comparison with the feed intake reflected enhanced feed (milk) efficiency. Many experiments [8,13] observed a positive relationship between MSP supplementation and enzymatic treatments and milk production. The use of probiotics has been observed to improve microbial ecology, feed conversion ratio, and nutrient intake, resulting in better performance [9]. The supplementation with MSP causes some changes in ruminal bacterial community composition, including bacteria in the family of Lactobacillales [9], which plays a vital role in stabilizing ruminal pH [24]. The cumulative effect of greater nutrient intakes and digestibility and improved ruminal fermentation (i.e., propionate concentration) may be considered the main reasons for greater daily milk. Additionally, higher ruminal propionate concentration, which is a precursor for glucose and lactose synthesis, has favorable effects on milk yield as propionate appears to augment energy availability [37]. As earlier mentioned, greater blood glucose suggests a good energy status of animals, and can be another reason for increases in milk production in the DPL-fed ewes [37].

Multi-species probiotics treatments showed higher concentration of milk lactose compared to enzyme treatment, but the reason is unclear in this study. It is well-known that lactose concentration depends on nutrient digestibility (especially OM and NSC) and ruminal propionate concentration, and all of them followed the same trends in both fibrolytic enzymes- and MSP-treated diets. Nutritional factors contribute about 50% of the variations in milk composition and yields [38]. Fibrolytic enzymes and MSP treatments increased the concentrations of milk fats and milk energy, which may be attributed to the increased ruminal acetate concentration because of enhanced fiber digestion with DPL treatments. Ruminal acetate is the major precursor for mammary gland fatty-acid synthesis [38].

Treatments had minimal effects on individual fatty acids; however, fibrolytic enzymes and MSP treatments increased the concentrations of C18:1n9 *tran*s, C18:2 trans-10, cis-12, C18:3n3, C18.3n6, C20:5n3, PUFA, total CLA and UFA:SFA ratio, but decreased the atherogenicity index. More than half of milk fatty acids arise from plasma uptake and the rest are synthesized in mammary glands [39]. As previously noted, enzymes and MSP treatments improved fiber digestion, which might be associated with altered milk fatty-acid profiles as a result of changes in the acetate-to-propionate ratio in the rumen.

The increased PUFA concentration and UFA:SFA ratio suggest that DPL treatments affected the ruminal bacterial activities responsible for biohydrogenation of dietary PUFA [38]. Minor improvements in a few n-3 FA contents in milk were also noted due to probiotic or enzyme treatments. It is well-documented that PUFA concentrations in milk depend mainly on the amount absorbed in the small intestine [38] as a result of escaping of ruminal biohydrogenation, which makes them available for incorporation into milk fat. Milk CLA are produced in the rumen when linoleic acid is partially biohydrogenated by ruminal bacteria. In addition, CLA is synthesized in the mammary glands by desaturation of rumenic acid (a partial hydrogenation product of linoleic acid) contributing about 70% of total milk CLA [40]. The extracts of DPL were found to contain many phytochemicals, including polyphenols, flavonoids, tannins, saponins, and quinines with antimicrobial activities [41]. Ensiling with MSP or enzyme may release more phenolic and saponin compounds from DPL, and these compounds can reduce ruminal microbial biohydrogenation of UFA that may be absorbed from intestine to blood and subsequently to milk increasing PUFA and CLA contents in meat and milk. Greater n-3 FA concentrations in milk have also been reported due to lactobacillus probiotic feeding to goats [42]. Increased proportion of PUFA and CLA in milk caused by treated DPL would be beneficial for human health.

## 5. Conclusions

Ensiling of date-palm leaves with fibrolytic enzymes or MSP before feeding to lactating Farafra ewes increased feed intake, improved nutrient digestibility, positively altered ruminal fermentation, and improved lactational performance and milk nutritive value (milk fatty acid profile) compared to the date-palm leaves ensiled without additives. Fibrolytic enzymes-treated DPL at 20% of diet improved fiber digestibility, while DPL at 40% increased milk production and feed efficiency compared to other treatments. Date-palm leaves ensiled with MSP or fibrolytic enzymes with minor differences between treatments may be used to improve milk production performance and milk quality in ewes under arid conditions. Fibrolytic enzyme treatment is recommended over MSP treatment.

## Figures and Tables

**Table 1:** Chemical composition of ingredients and diets (g/kg DM).

	Ingredient ^1^				Diet ^2^				
Item	CFM	DPL-No Additives ^3^	DPL-ENZ ^4^	DPL-MSP ^5^	Control	ENZ50	ENZ100	MSP50	MSP100
DM	903.2	741.0	754.0	762.0	838.3	840.9	843.5	842.5	846.7
OM	922.9	907.0	908.0	911.0	916.5	916.7	916.9	917.3	918.1
CP	165.0	47.0	50.0	50.0	117.8	118.4	119.0	118.4	119.0
EE	46.8	22.0	21.0	22.0	36.9	36.7	36.5	36.9	36.9
NSC	414.0	276.0	346.0	278.0	358.8	372.8	386.8	359.2	369.6
NDF	297.1	562.0	491.0	561.0	403.1	388.9	374.7	402.9	402.7
ADF	175.1	316.0	297.0	319.0	231.5	227.7	223.9	232.1	232.7
Lignin	33.0	122.0	125.0	121.0	68.6	69.2	69.8	68.4	68.2
Cellulose ^6^	142.0	194.0	172.0	198.0	162.8	158.4	154.0	163.6	164.4
Hemicellulose ^6^	122.0	246.0	194.0	242.0	171.6	161.2	150.8	170.8	170.0

ADF, acid detergent fiber; CP, crude protein; DM, dry matter; EE, ether extract; NDF, neutral detergent fiber; NSC, non-structural carbohydrates; OM, organic matter. ^1^ Ingredients: CFM, concentrate feed mixture; DPL-no additives, date palm leaves ensiled without additives; DPL-ENZ, date palm leaves ensiled with fibrolytic enzymes; DPL-MSP, date palm leaves ensiled with MSP. ^2^ Diet: Control diet contained 600 g of concentrate feed mixture and 400 g of date palm leaves (DPL) ensiled without additives or the ensiled DPL without additive was replaced with DPL ensiled with fibrolytic enzymes (ENZ50 and ENZ100) or MSP (MSP50 and MSP100) at 50 or 100% level, respectively. ^3^ pH = 4.3, ammonia-N = 64 g/kg of total N, volatile fatty acids = 73.0 g/kg DM, aflatoxin F_1_ = 3.2 µg/kg of DM. ^4^ pH = 3.8, ammonia-N = 51 g/kg of total N, volatile fatty acids = 89.0 g/kg DM, aflatoxin F_1_ = 3.0 µg/kg of DM. ^5^ pH = 4.1, ammonia-N = 44 g/kg of total N, volatile fatty acids = 86.0 g/kg DM, aflatoxin F_1_ = 3.0 µg/kg of DM. ^6^ Calculated values (cellulose = ADF-lignin, hemicellulose = NDF-ADF).

**Table 2:** Intake and nutrient digestibility of diets containing date-palm leaves ensiled without additives or ensiled with fibrolytic enzymes or MSP and fed to lactating Farafra ewes ^1^.

Item		Enzyme (ENZ)		Multi-Species Probiotics (MSP)		SEM	*p*-ENZ		*p*-MSP		*p*-ENZ vs. MSP
	Control	ENZ50	ENZ100	MSP50	MSP100		Linear	Quadratic	Linear	Quadratic	
Intake (g/d)											
Date palm leaves	309	331	336	331	336	2.4	<0.001	0.002	<0.001	0.005	0.895
Total ^2^	819	841	846	841	846	2.4	<0.001	0.002	<0.001	0.005	0.895
Digestibility (g/kg DM)											
DM	561	601	607	626	623	5.6	<0.001	0.011	<0.001	<0.001	0.001
OM	562	615	625	622	624	6.2	<0.001	0.005	<0.001	0.001	0.593
CP	553	608	620	623	625	4.9	<0.001	0.001	<0.001	<0.001	0.400
EE	581	611	629	611	618	5.3	<0.001	0.332	<0.001	0.075	0.263
NSC	557	621	622	610	607	4.7	<0.001	<0.001	<0.001	<0.001	0.005
NDF	520	569	574	553	571	5.7	<0.001	0.002	<0.001	0.272	0.093
ADF	515	563	559	554	559	4.1	<0.001	<0.001	<0.001	0.002	0.299
Cellulose	528	576	573	562	572	3.3	<0.001	<0.001	<0.001	0.005	0.029
Hemicellulose	524	570	564	558	563	2.7	<0.001	<0.001	<0.001	<0.001	0.223

SEM, standard error of the mean. ADF, acid detergent fiber; CP, crude protein; DM, dry matter; EE, ether extract; NDF, neutral detergent fiber; NSC, non-structural carbohydrates; OM, organic matter. ^1^ Diet: Control diet contained 600 g of concentrate feed mixture and 400 g of date-palm leaves (DPL) ensiled without additives, or the DPL ensiled without additives was replaced with DPL ensiled with fibrolytic enzymes (ENZ50 and ENZ100) or MSP (MSP50 and MSP100) at 50 or 100% level, respectively. ^2^ All ewes were fed the same amounts of concentrates (510 g DM/ewe/d).

**Table 3:** Ruminal fermentation of lactating Farafra ewes fed diets containing date-palm leaves ensiled without additives or ensiled with fibrolytic enzymes or MSP ^1^.

Item		Enzyme (ENZ)		Multi-Species Probiotics (MSP)		SEM	*p*-ENZ		*p*-MSP		*p*-ENZ vs. MSP
	Control	ENZ50	ENZ100	MSP50	MSP100		Linear	Quadratic	Linear	Quadratic	
pH	5.71	5.64	5.64	5.62	5.66	0.141	0.604	0.093	0.111	0.215	0.317
Ammonia-N, mg/dL	30.1	32.9	32.3	33.6	33.2	0.37	<0.001	0.001	<0.001	<0.001	0.029
Total volatile fatty acids, mmol/L	121	133	135	134	135	1.4	<0.001	0.001	<0.001	0.001	0.774
Acetate, mmol/L	71.9	79.7	80.7	81.2	81.2	0.95	<0.001	0.004	<0.001	0.001	0.280
Propionate, mmol/L	27.2	30.8	31.7	30.7	31.3	0.51	<0.001	0.034	<0.001	0.023	0.597
Butyrate, mmol/L	21.6	22.9	22.1	21.8	22.5	0.75	0.620	0.267	0.382	0.748	0.635
Acetate:propionate ratio	2.67	2.61	2.56	2.68	2.62	0.05	0.146	0.978	0.528	0.591	0.222

SEM, standard error of the mean. ^1^ Diet: Control diet contained 600 g of concentrate feed mixture and 400 g of date palm leaves (DPL) ensiled without additives, or the DPL ensiled without additives was replaced with DPL ensiled with fibrolytic enzymes (ENZ50 and ENZ100) or MSP (MSP50 and MSP100) at 50 or 100% level, respectively.

**Table 4:** Blood measurements of lactating Farafra ewes fed diets containing date-palm leaves ensiled without additives or ensiled with fibrolytic enzymes or MSP ^1^.

Item		Enzyme (ENZ)		Multi-Species Probiotics (MSP)		SEM	*p*-ENZ		*p*-MSP		*p*-ENZ vs. MSP
	Control	ENZ50	ENZ100	MSP50	MSP100		Linear	Quadratic	Linear	Quadratic	
Total proteins, g/dL	7.23	7.56	7.58	7.62	7.62	0.033	<0.001	0.001	<0.001	<0.001	0.145
Albumin, g/dL	3.86	4.06	4.07	4.11	4.10	0.018	<0.001	<0.001	<0.001	<0.001	0.059
Globulin, g/dL	3.37	3.50	3.51	3.51	3.53	0.135	0.506	0.158	0.482	0.139	0.656
Urea-N, mg/dL	39.1	38.8	38.7	39.3	39.3	0.26	0.333	0.777	0.478	0.684	0.029
Glucose, mg/dL	77.1	84.0	84.2	84.9	84.9	0.24	<0.001	<0.001	<0.001	<0.001	0.801
ALT, units/L	15.6	16.0	15.5	16.0	16.0	0.26	0.773	0.114	0.268	0.566	0.389
AST, units/L	32.8	32.2	31.9	31.4	31.2	0.91	0.666	0.508	0.251	0.513	0.111
Triglycerides, mg/dL	164	169	171	171	172	1.5	0.003	0.634	0.001	0.114	0.245
HDL, mg/dL	93.6	94.0	94.1	94.8	95.2	0.43	0.398	0.662	0.007	0.446	0.130
LDL, mg/dL	71.0	70.6	72.1	72.1	71.3	0.57	0.182	0.187	0.699	0.175	0.527
Antioxidant capacity, mg/dL	98.8	110.0	110.0	110.6	111.1	1.61	<0.001	0.006	<0.001	0.005	0.606
BHBA, mg/dL	0.84	0.84	0.87	0.91	0.89	0.019	0.244	0.699	0.046	0.056	0.022
NEFA, mg/dL	1.73	1.74	1.75	1.81	1.79	0.053	0.788	0.918	0.447	0.454	0.298

SEM, standard error of the mean. BHB, beta-hydroxybutyrate; AST, aspartate aminotransaminase; ALT, alanine aminotransaminase; HDL, high-density lipoprotein cholesterol; LDL, low-density lipoprotein cholesterol; NEFA, nonesterified fatty acids. ^1^ Diet: Control diet contained 600 g of concentrate feed mixture and 400 g of date palm leaves (DPL) ensiled without additives, or the DPL ensiled without additives was replaced with DPL ensiled with fibrolytic enzymes (ENZ50 and ENZ100) or MSP (MSP50 and MSP100) at 50 or 100% level, respectively.

**Table 5:** Milk production, composition, and feed efficiency of lactating Farafra ewes fed diets containing date-palm leaves ensiled without additives or ensiled with fibrolytic enzymes or MSP ^1^.

Item		Enzyme (ENZ)		Multi-Species Probiotics (MSP)		SEM	*p*-ENZ		*p*-MSP		*p*-ENZ vs. MSP
	Control	ENZ50	ENZ100	MSP50	MSP100		Linear	Quadratic	Linear	Quadratic	
Production, g/d (unless stated otherwise)											
Milk	603	657	667	652	660	13.3	0.001	0.182	0.003	0.209	0.645
Energy corrected milk (ECM)	637	734	748	730	742	15.2	<0.001	0.027	<0.001	0.029	0.750
Fat corrected milk (4% FCM)	617	713	729	704	713	14.5	<0.001	0.024	<0.001	0.028	0.407
Milk energy output, MJ/d	1.96	2.27	2.31	2.26	2.30	0.047	<0.001	0.024	<0.001	0.024	0.801
Total solids	80.2	91.5	93.0	91.4	92.9	1.88	<0.001	0.036	<0.001	0.037	0.938
Solids non-fat	55.1	61.5	62.3	61.8	62.9	1.29	0.001	0.082	<0.001	0.081	0.702
Fat	25.0	30.0	30.8	29.6	30.0	0.62	<0.001	0.006	<0.001	0.007	0.306
Protein	25.0	27.4	27.8	27.2	27.9	0.62	0.002	0.203	0.002	0.298	0.939
Lactose	25.5	28.9	29.3	29.5	30.0	0.59	<0.001	0.033	<0.001	0.015	0.279
Ash	4.61	5.17	5.22	5.07	5.12	0.120	0.001	0.089	0.004	0.164	0.412
Composition, g/kg unless stated otherwise											
Total solids	133	139	140	140	141	0.4	<0.001	<0.001	<0.001	<0.001	0.808
Solids non-fat	91.5	93.6	93.3	94.8	95.4	0.30	<0.001	0.001	<0.001	0.001	<0.001
Fat	41.6	45.7	46.2	45.4	45.4	0.30	<0.001	<0.001	<0.001	<0.001	0.055
Protein	41.5	41.7	41.6	41.7	42.2	0.23	0.818	0.690	0.051	0.692	0.178
Lactose	42.3	44.1	43.9	45.3	45.4	0.17	<0.001	<0.001	<0.001	<0.001	<0.001
Ash	7.65	7.87	7.83	7.80	7.77	0.120	0.248	0.341	0.423	0.498	0.584
Milk energy content, MJ/kg	3.25	3.45	3.47	3.46	3.48	0.012	<0.001	<0.001	<0.001	<0.001	0.347
Feed efficiency											
Milk:intake ratio	0.74	0.78	0.79	0.78	0.78	0.016	0.021	0.407	0.040	0.356	0.691
ECM:intake ratio	0.78	0.87	0.88	0.87	0.88	0.018	<0.001	0.073	0.001	0.073	0.805
FCM:intake ratio	0.76	0.85	0.86	0.84	0.84	0.017	<0.001	0.064	0.001	0.069	0.478

SEM, standard error of the mean. ^1^ Diet: Control diet contained 600 g of concentrate feed mixture and 400 g of date-palm leaves (DPL) ensiled without additives, or the DPL ensiled without additive was replaced with DPL ensiled with fibrolytic enzymes (ENZ50 and ENZ100) or MSP (MSP50 and MSP100) at 50 or 100% level, respectively.

**Table 6:** Milk fatty acid (FA) profile (g/100 g FA) of lactating Farafra ewes fed diets containing date-palm leaves ensiled without additives or ensiled with fibrolytic enzymes or MSP ^1^.

Item		Enzyme (ENZ)		Multi-Species Probiotics (MSP)		SEM	*p*-ENZ		*p*-MSP		*p*-ENZ vs. MSP
	Control	ENZ50	ENZ100	MSP50	MSP100		Linear	Quadratic	Linear	Quadratic	
C4:0	2.82	2.79	2.73	2.90	2.77	0.063	0.306	0.908	0.560	0.166	0.213
C6:0	2.05	2.09	2.11	2.07	2.11	0.036	0.240	0.747	0.259	0.802	0.689
C8:0	2.27	2.32	2.34	2.32	2.35	0.096	0.507	0.426	0.342	0.586	0.782
C10:0	5.07	5.03	5.11	5.12	5.12	0.035	0.441	0.144	0.407	0.552	0.187
C11:0	0.88	0.90	0.89	0.91	0.92	0.014	0.544	0.529	0.059	0.660	0.150
C12:0	3.21	3.22	3.22	3.27	3.26	0.026	0.762	0.746	0.124	0.219	0.063
C14:0	9.09	8.97	8.92	9.13	9.10	0.056	0.034	0.662	0.857	0.617	0.083
C14:1	0.67	0.68	0.68	0.69	0.69	0.008	0.105	0.426	0.111	0.244	0.044
C15:0	0.54	0.54	0.55	0.56	0.57	0.005	0.742	0.684	0.061	0.913	0.091
C16:0	26.3	25.0	24.9	24.3	24.7	0.95	0.111	0.572	0.481	0.601	0.594
C16:1	1.20	1.23	1.24	1.30	1.31	0.068	0.473	0.405	0.691	0.661	0.501
C17:0	0.89	0.89	0.89	0.94	0.94	0.027	0.885	0.933	0.528	0.931	0.351
C18:0	16.5	16.2	16.3	16.4	16.2	0.09	0.072	0.131	0.227	0.854	0.324
C18:1n9*cis*	24.4	25.7	25.6	25.5	25.3	0.84	0.222	0.530	0.771	0.905	0.068
C18:1n9*trans*	2.42	2.79	2.88	2.89	2.89	0.296	0.001	0.076	0.019	0.225	0.068
C18:2 *trans*-10, *cis*-12	0.28	0.30	0.30	0.31	0.31	0.005	0.001	0.035	<0.001	0.011	0.119
C18:2 *cis*-9, *trans*-11	0.17	0.20	0.19	0.21	0.19	0.011	0.245	0.400	0.281	0.040	0.510
C18:3n3	0.16	0.18	0.18	0.18	0.18	0.003	0.001	0.026	0.001	0.013	0.750
C18:3n6	0.36	0.39	0.39	0.40	0.40	0.004	<0.001	0.001	<0.001	0.001	0.183
C20:0	0.68	0.64	0.63	0.64	0.64	0.066	0.231	0.066	0.071	0.098	0.259
C20:5n3	0.15	0.17	0.18	0.18	0.17	0.004	<0.001	0.216	0.001	0.001	0.964
C22:5n3	0.19	0.21	0.22	0.23	0.22	0.005	<0.001	0.045	0.001	0.001	0.431
SFA	70.3	68.5	68.6	68.5	68.7	0.94	0.661	0.601	0.089	0.088	0.516
UFA	30.0	31.8	31.9	31.9	31.6	0.94	0.568	0.083	0.081	0.024	0.482
MUFA	28.7	30.4	30.4	30.4	30.2	0.94	0.056	0.082	0.111	0.146	0.376
PUFA	1.32	1.46	1.46	1.50	1.47	0.014	<0.001	0.001	<0.001	<0.001	0.074
Total CLA	0.45	0.50	0.49	0.52	0.50	0.012	0.011	0.110	0.003	0.004	0.224
n6: n3 FA ratio	2.20	2.22	2.22	2.22	2.26	0.045	0.662	0.867	0.300	0.949	0.620
UFA: SFA ratio	0.43	0.46	0.46	0.47	0.46	0.003	<0.001	<0.001	<0.001	<0.001	0.700
Atherogenicity index ^2^	2.20	2.01	2.00	2.01	2.04	0.015	<0.001	<0.001	<0.001	<0.001	0.287

SEM, standard error of the mean. CLA, conjugated linoleic acid (C18:2 *trans*-10, *cis*-12 and C18:2 *cis*-9, *trans*-11); MUFA, mono unsaturated fatty acids; PUFA, poly unsaturated fatty acids; SFA, saturated fatty acids; UFA, unsaturated fatty acids. ^1^ Diet: Control diet contained 600 g of concentrate feed mixture and 400 g of date-palm leaves (DPL) ensiled without additives, or the DPL was replaced with DPL ensiled with fibrolytic enzymes (ENZ50 and ENZ100) or MSP (MSP50 and MSP100) at 50 or 100% level, respectively. ^2^ Calculated according to Ulbricht and Southgate [19]: Atherogenicity index = (C12:0 + 4 × C14:0 + C16:0)/∑ of UFA.

## Data Availability

The datasets generated during and/or analyzed during the current study are available from the corresponding author on reasonable request.
